# Effect of Repetitive Lysine-Tryptophan Motifs on the Eukaryotic Membrane

**DOI:** 10.3390/ijms14012190

**Published:** 2013-01-22

**Authors:** Ramamourthy Gopal, Jong Kook Lee, Jun Ho Lee, Young Gwon Kim, Gwang Chae Oh, Chang Ho Seo, Yoonkyung Park

**Affiliations:** 1 Research Center for Proteineous Materials, Chosun University, Gwangju 501-759, Korea; E-Mails: ramagopa@gmail.com (R.G.); seal9669@hanmail.net (J.K.L.); 2 Department of Biotechnology, Chosun University, Gwangju 501-759, Korea; E-Mails: juno6267@hanmail.net (J.H.L.); kyg1022@hanmail.net (Y.G.K.); 3 Department of Bioinformatics, Kongju National University, Kongju 314-701, Korea; E-Mails: gcoh@gjtp.or.kr (G.C.O.); chseo@kongju.ac.kr (C.H.S.)

**Keywords:** (KW)_5_, hemolytic peptide, eukaryotic membrane, phosphatidylcholine, cholesterol, sphingomyelin, aggregation

## Abstract

In a previous study, we synthesized a series of peptides containing simple sequence repeats, (KW)*_n_–*NH_2_ (*n* = 2,3,4 and 5) and determined their antimicrobial and hemolytic activities, as well as their mechanism of antimicrobial action. However, (KW)_5_ showed undesirable cytotoxicity against RBC cells. In order to identify the mechanisms behind the hemolytic and cytotoxic activities of (KW)_5_, we measured the ability of these peptides to induce aggregation of liposomes. In addition, their binding and permeation activities were assessed by Trp fluorescence, calcein leakage and circular dichrorism using artificial phospholipids that mimic eukaryotic liposomes, including phosphatidylcholine (PC), PC/sphingomyelin (SM) (2:1, *w*/*w*) and PC/cholesterol (CH) (2:1, *w*/*w*). Experiments confirmed that only (KW)_5_ induced aggregation of all liposomes; it formed much larger aggregates with PC:CH (2:1, *w*/*w*) than with PC or PC:SM (2:1, *w*/*w*). Longer peptide (KW)_5_, but not (KW)_3_ or (KW)_4_, strongly bound and partially inserted into PC:CH compared to PC or PC:SM (2:1, *w*/*w*). Calcein release experiments showed that (KW)_5_ induced calcein leakage from the eukaryotic membrane. Greater calcein leakage was induced by (KW)_5_ from PC:CH than from PC:SM (2:1, *w*/*w*) or PC, whereas (KW)_4_ did not induce calcein leakage from any of the liposomes. Circular dichroism measurements indicated that (KW)_5_ showed higher conformational transition compared to (KW)_4_ due to peptide-liposome interactions. Taken together, our results suggest that (KW)_5_ reasonably mediates the aggregation and permeabilization of eukaryotic membranes, which could in turn explain why (KW)_5_ displays efficient hemolytic activity.

## 1. Introduction

Native and synthetic antimicrobial peptides (AMPs) exhibited a broad spectrum of activities against antibiotic resistant bacterial strains, including cancer cells [1]. Moreover, AMPs constitute key parts of the human and mammalian innate immune systems. In addition, AMPs are also involved in multiple defensive roles, such as regulating the inflammatory response and chemo-attracting cells of the adaptive immune system to wound/infection sites, binding and neutralizing lipopolysaccharides and promote re-epithelialization and wound closure in human skin against bacterial infections [2,3]. However, synthetic peptides utility is currently limited to topical application, because of their cytotoxicity against the eukaryotic cell membranes.

Prokaryotic and eukaryotic cell membranes are mainly composed of phospholipids that differentiate their compositions [4]. It is well known that eukaryotic cell membranes are mainly composed of electrically neutral zwitterionic phospholipids, including phosphatidylcholine (PC), cholesterol (CH) and sphingomyelin (SM) [5]. Among these three phospholipids, the percentage of PC in the outer leaflets of eukaryotic membranes is higher than that of CH or SM [6]. Indeed, the outer leaflets of eukaryotic membranes are composed of lipids with no net charge. Accordingly, the small amount of negatively charged lipids is essentially located in the inner leaflet [7]. In contrast, bacterial membranes (e.g., in *E. coli*) lack CH, contain phosphatidylethanolamine as their most common zwitterionic lipid and are rich in anionic lipids, such as phosphatidylglycerol, and cardiolipin in their outer membranes [8]. This differentiation in phospholipid content between prokaryotic and eukaryotic membranes promotes selectivity by AMPs [9,10]. This selectivity of AMPs towards bacterial plasma membranes is very important for the design of novel antibiotic peptides with low cytotoxicity. However, many AMPs are non-selective between mammalian and microbial cells (e.g., pardaxin [11], melittin [12] and cathelicidins [13]). Therefore, various studies have attempted to determine the parameters that are responsible for AMP toxicity. It has been determined that high helicity, hydrophobicity and amphipathicity are correlated with an increase in eukaryotic cell toxicity [14]. Recent studies have also shown that aggregation/oligomeration of cationic antimicrobial peptides (cAMPs) is another parameter that is important for pore formation, regardless of the mechanism of cytotoxicity [15,16]. Accordingly, efforts have been undertaken to improve the selectivity of cAMPs, including sequence modification, to optimize their physiochemical parameters [17–20]. At present, short AMPs that minimize damage to host cells or tissue appear to be the most promising candidates for large-scale production [21,22]. In addition, the large size of AMPs is a hindrance due to high manufacturing costs. In response, selective short AMPs are currently being developed based on their amino acid composition, charge, hydrophobicity and length [21–25]. Specifically, lysine (K) or arginine (R)-tryptophan (W or Trp) or isoleucine (I)-phenylalanine (F) motifs have been designed for bacterial membrane selectivity [22,26]. However, KW, RIF or RW series peptides show increased hemolytic activity and hydrophobicity with higher chain length [22,26]. In a separate study using a *de novo* designed alpha helical HDP, high hydrophobicity was found to correlate with strong hemolytic activity [27]. However, there is insufficient data to support this claim. While hemolytic activity correlates with hydrophobicity values, there could be other factors affecting hemolytic activity. Therefore, we investigated the effects of peptide on model vesicles composed of a mixture of PC, CH and SM in order to determine their molecular mechanisms, since these three lipids are major constituents of most mammalian plasma membranes. It is also important to note that CH effectively suppressed the toxicity of AMPs against the eukaryotic membrane by preventing peptide induced eukaryotic membrane disruption via a barrel stave mechanism or other mechanism [28,29]. Also, a variety of biophysical approaches have reported mechanisms of membrane permeation/disruption activities of AMPs, including AMPs-induced liposome aggregation [30–33]. However, we believe this study will be useful in understanding the role of PC, SM and CH on hemolytic activity of AMPs. We found that only (KW)_5_ showed a strong interaction towards PC, PC:SM (2:1, *w*/*w*) and PC:CH (2:1, *w*/*w*) of the erythrocyte membrane. Moreover, (KW)_5_ induced aggregation/permeabilization of eukaryotic membranes.

## 2. Results and Discussion

Several studies have clearly shown that AMPs demonstrate activity against negatively charged bacterial membranes, whereas AMPs do not show strong activity towards zwitterionic eukaryotic membranes, suggesting non-activity against human cells [34–40]. Similarly, (KW)_4_ has shown strong selectivity against bacterial over eukaryotic membranes based on membrane interactions. However, when chain length exceeds the optimum number of 10 residues, (KW)_5_ shows interactions with both bacterial and eukaryotic membranes, resulting in antibacterial activity with cytoxicity towards eukaryotic cells [26]. In the present study, we investigated the interactions of (KW)*_n_* peptides with eukaryotic membranes to determine the increased hemolytic mechanism of (KW)_5_ compared to (KW)_4_.

### 2.1. Liposomes Aggregation

**Figure 1:**
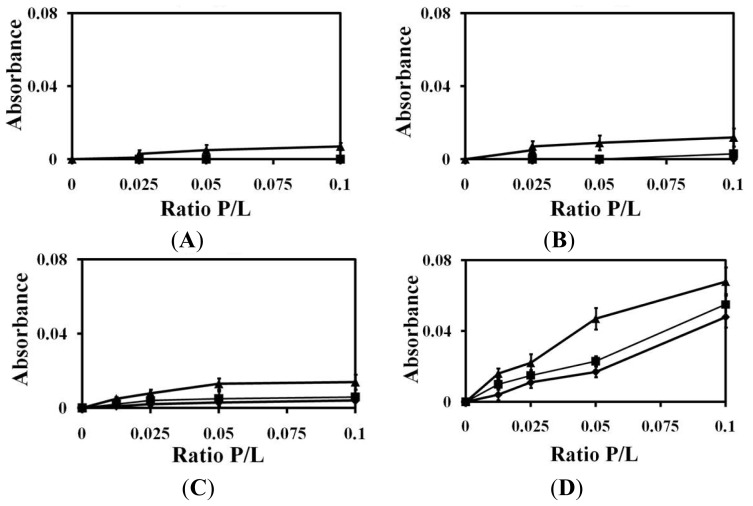
Large unilamellar vesicles (LUV) aggregation. Solutions containing various concentrations of peptide were added to a suspension of 400 μM PC (diamonds), PC:SM (2:1, *w*/*w*) (squares) and PC:CH (2:1, *w*/*w*) (triangles), after which aggregation was monitored based on changes in the absorbance of LUVs at 405 nm. (**A**) (KW)_2_; (**B**) (KW)_3_; (**C**) (KW)_4_; (**D**) (KW)_5_.

Liposome aggregation showed peptide-lipid interactions as previously reported [26,41]. We measured changes in liposome turbidity in order to determine the effects of (KW)*_n_* peptides on the sizes of PC, PC:SM (2:1, *w*/*w*) and PC:CH (2:1, *w*/*w*) vesicles (Figure 1). We found that (KW)_2_, (KW)_3_ and (KW)_4_ did not induce liposome aggregation towards PC, PC:SM (2:1, *w*/*w*) or PC:CH (2:1, *w*/*w*) vesicles, even at larger peptide concentrations. However, our results showed that (KW)_5_ was able to induce aggregation of liposomes. In addition, (KW)_5_ induced larger aggregation of PC:CH than either 100% PC or PC:SM (2:1, *w*/*w*). This could be attributed to the membrane behavior of CH leading to enhanced peptide accumulation in the hydrophobic core of its bilayers, resulting in increased liposome aggregation. It was reported that (KW)_5_ is soluble and weakly self-aggregates in PBS, as determined from Trp fluorescence spectra, showing W-residues to be in a less polar environment (λ_max_ = 342) [26]. Other studies have proposed that a peptide’s aggregation state in solution might affect eukaryotic membranes, resulting in cytotoxicity against RBCs [42,43]. This is in accordance with previous data on the hemolytic activity of (KW)_5_ peptide [26].

### 2.2. Characterization of Trp Environment Using Fluorescence Spectroscopy

**Figure 2:**
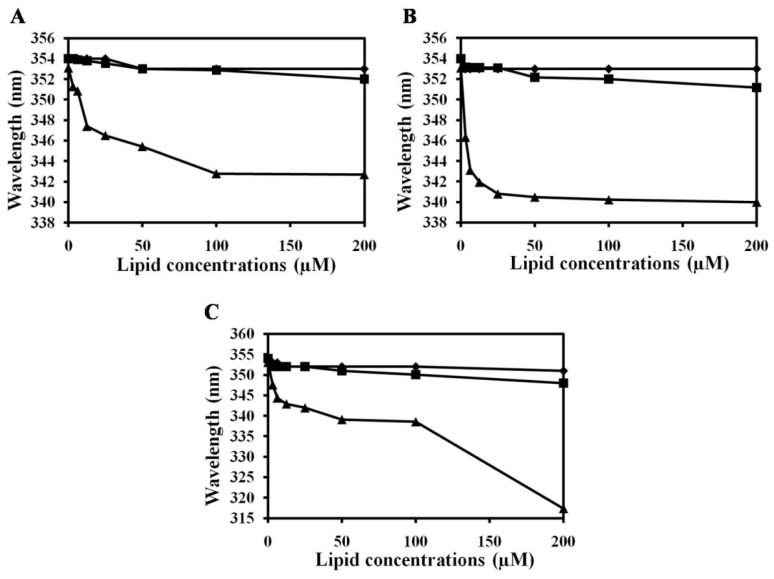
Blue shift in Trp fluorescence. Emission maxima from Trp in peptides in the presence of (**A**) PC; (**B**) PC:SM (2:1, *w*/*w*); and (**C**) PC:CH (2:1, *w*/w). (KW)_3_ (diamonds), (KW)_4_ (squares), (KW)_5_ (triangles).

To determine the mechanism behind (KW)_5_-induced aggregation of eukaryotic liposomes and whether or not it is mediated by interactions with the zwitterionic eukaryotic membrane, Trp fluorescence blue shift and Trp quenching experiments were carried out to compare the membrane-binding affinities of the peptides. Peptide binding to lipid bilayers was examined by recording the Trp fluorescence emission spectra in the presence of vesicles composed of zwitterionic PC, PC:SM (2:1, *w*/*w*) and PC:CH (2:1, *w*/*w*) (Figure 2). Blue shift assay indicated that (KW)_3_ and (KW)_4_ did not strongly interact with any of the liposomes. Peptides that interact with negatively charged bacterial membranes through electrostatic interactions initially experience enhanced accumulation on the bilayer surface [26]. On the other hand, bilayer disruption strongly depends on the properties of lipids, as well as peptide charge/hydrophobicity. Both (KW)_3_ and (KW)_4_ did not strongly bind with PC, PC:SM (2:1, *w*/*w*) or PC:CH (2:1, *w*/*w*) due to their zwitterionic character. Since PC membranes lack a net negative charge, cationic peptides cannot undergo electrostatic interactions with them. In contrast, (KW)_5_ showed strong interactions towards all liposomes. Among the three liposomes, larger blue shift of (KW)_5_ peptide suggests that its Trp side chain partitions more into PC:CH (7:3, *w*/*w*) lipid bilayers.

The accessibility of Trp residues into lipid bilayers can be determined by measuring the Stern-Volmer quenching constant for each peptide using soluble acrylamide as a quencher. In buffer, these peptides are almost completely accessible by acrylamide in aqueous solution [26]. The *K*_SV_ of (KW)_5_ peptide was 1.7 M^−1^, indicating that the Trp residues of (KW)_5_ were more protected in the presence of PC:CH (2:1, *w*/*w*) vesicles than in PC or PC:SM (2:1, *w*/*w*) vesicles (Table 1). This tendency was consistent with its potent aggregation activity towards eukaryotic liposomes. The lower *K*sv values of vesicles composed of PC, PC:SM (2:1 *w*/*w*) and PC:CH (2:1 *w*/*w*) could be attributed to the hydrophobic effects and van der Waals forces that likely dominate the interactions between neutral lipids and the hydrophobic residues of (KW)_5_.

### 2.3. Calcein Leakage

**Figure 3:**
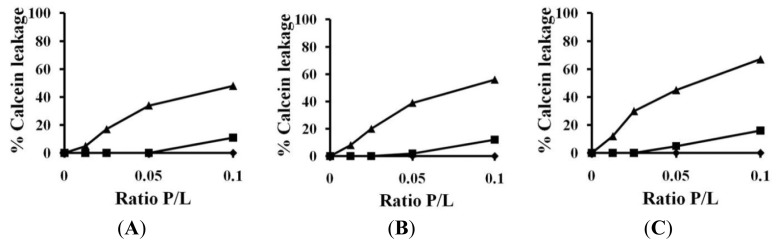
Percent leakage of calcein from (**A**) PC; (**B**) PC:SM (2:1, *w*/*w*); and (**C**) PC:CH (2:1, *w*/*w*) was measured for 25 min after the addition of various doses of peptide.

Calcein leakage assay was performed to investigate interactions between AMPs and model liposomes [44]. We determined the ability of these peptides to cause leakage of entrapped calcein from PC, PC:SM (2:1 *w*/*w*) and PC:CH (2:1 *w*/*w*) vesicles, which mimic eukaryotic membranes. As shown in Figure 3, (KW)_2_ and (KW)_3_ did not release calcein from any of the three liposomes, whereas (KW)_4_ showed slight calcein leakage at a higher concentration of 64 μm. Generally, significant leakage is not observed from LUVs enriched in PC, CH and SM by (KW)_2_, (KW)_3_ or (KW)_4_, which is consistent with their inability to induce aggregation/insertion in LUVs. In fact, Trp has a preference for the interfacial region of the lipid bilayer [45,46]. However, (KW)_2_, (KW)_3_ and (KW)_4_ did not deeply penetrate the hydrophobic region of the lipid bilayer, compared to (KW)_5_ (Table 1). This may suggest that interactions of cationic peptides, such as (KW)_2_, (KW)_3_ and (KW)_4_, with neutral zwitterionic lipids are weakened due to repulsion between the cationic peptides and positively charged head group of amino choline in PC, thereby inhibiting the hydrophobic interactions between peptides and eukaryotic membranes. However, (KW)_5_ showed eukaryotic membrane disruption ability due to two reasons. Firstly, (KW)_5_ demonstrates weak aggregation in aqueous solution, which facilitates association of the peptide with liposomes by overcoming the repulsion between the peptide and lipid head group. Secondly, it has been described that increasing the number of Trp residues in AMPs naturally enhances membrane interactions and liposome leakage from PC or PC:CH; thus, (KW)_5_ is more able to interact with the interfacial region of the bilayer [47]. Previous studies have also described AMP activity on PC vesicle through interfacial activity, resulting in binding, insertion and perturbation [48]. Interfacial activity is also displayed by peptides having sufficient hydrophobicity [49]. It has been clearly suggested that (KW)_5_ peptides has higher hydrophobicity compared to (KW)_3_ and (KW)_4_. (KW)_5_ peptide associates with the hydrocarbon core of neutral bilayers and localizes near the aqueous phase in zwitterionic model membranes (Table 1). This increased local density of (KW)_5_, results in local disruption of lipid chains packing, leading to boundary disruption of lipid domains. The induced calcein release by (KW)_5_ was consistent with this notion.

### 2.4. Circular Dichroism (CD)

**Figure 4:**
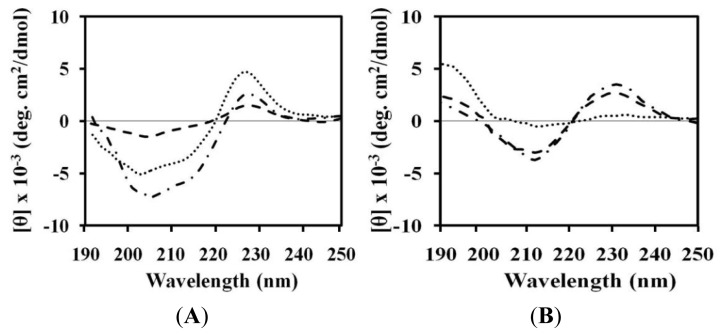
CD spectra for (KW)_4_ and (KW)_5_ peptides in the presence of PC (dashed line), PC:SM (2:1, *w*/*w*) (dashed dotted line) and PC:CH (2:1, *w*/*w*) (dotted). (**A**) (KW)_4_ and (**B**) (KW)_5_.

Conformational changes of peptides were investigated by CD spectroscopy. Dramatic changes in the CD spectra occurred when (KW)_5_ was mixed with neutral lipids. In the presence of buffer, these peptides display random coil conformations [26]. When (KW)_5_ was added to PC, PC:SM (2:1, *w*/*w*) and PC:CH (2:1, *w*/*w*) liposomes, larger changes in CD spectra were observed, especially in PC:CH (2:1, *w*/*w*). (KW)_5_ shifted its minimum at 200 nm to 215 nm and gained a maximum at 195 nm, indicating that an extended beta-sheet structure was formed (Figure 4). Similar behaviors have been observed with AMPs, such as (RW)_5_ [22], suggesting that the high cost of partitioning peptide bonds into the membrane interface is a major driving force behind secondary structure formation in membrane environments [49–51]. Other studies also reported that peptides that are rich in aromatic residues bind well to the membrane interface [52]. In addition, previous information has shown that beta-sheet formation occurs upon induction of liposome aggregation by peptides [53]. Our CD measurement data also indicated that (KW)_5_ underwent a conformational transition from a random coil [26] to a beta-sheet structure. This was consistent with its ability to disrupt lipid bilayers and promote aggregation of liposomes. Other AMPs, such as (KW)_2_, (KW)_3_ and (KW)_4_, did not strongly bind to zwitterionic PC, PC:SM (2:1, *w*/*w*) or PC:CH (2:1, *w*/*w*) vesicles, which was consistent with their lack of hemolytic activity in erythrocytes [9,53,54]. In this case, no specific interaction with cholesterol was observed, which indicates low toxicity towards normal mammalian cells [55]. Indeed, certain melittin analogues fail to adopt appreciable secondary structures in the presence of a mammalian mimetic membrane, which could be associated with their failure to bind to and permeabilize hRBCs through a toroidal-pore mechanism resembling hemolytic activity [56].

In this work, we confirmed that (KW)_5_ induces membrane aggregation (or interaction) and membrane perturbation of vesicles composed of PC:CH (2:1, *w*/*w*), PC/SM (2:1, *w*/*w*) and PC. We further demonstrated that SM, CH and PC are not specifically required for the action of (KW)_5_. Unlike (KW)_5_, it was reported that some eukaryotic membrane active peptides are selective to the lipid composition of the model eukaryotic membrane [57,58]. Although the results of this study support the idea that the mechanism of (KW)_5_ cytotoxicity involves membrane interactions/permeabilization along with liposome aggregation, further investigations are needed to explain how the liposome aggregation/fusion process is mediated.

## 3. Experimental Section

### 3.1. Materials

Rink amide 4-methylbenzhydrylamine resin, fluoren-9-ylmethoxycarbonyl (Fmoc) amino acids and other reagents for peptide synthesis were purchased from Calibochem-Novabiochem (La Jolla, CA, USA). CH from porcine liver, egg yolk l-α-PC and SM were from Avanti Polar Lipids Co. (Alabaster, AL, USA). Calcein was obtained from Molecular Probes (Eugene, OR, USA). All other reagents were of analytical grade. Buffers were prepared using double distilled water (Millipore Co., Bedford, MA, USA).

### 3.2. Peptide Synthesis and Purification

Peptides were synthesized and purified as reported previously [26]. The purity of all peptides were found to be >95%.

### 3.3. Preparation of Large Unilamellar Vesicles (LUVs)

Large unilamellar vesicles (LUVs) were prepared by the freeze-thaw method [34,35]. Dry lipid films were resuspended in 1–2 mL of appropriate buffer by vortexing. LUVs were prepared by nine freeze-thaw cycles under liquid nitrogen and a water bath at 50 °C. After preparation of vesicles, suspensions were extruded 14 times through a 0.2 μM pore polycarbonate membrane. Lipid concentration was determined by standard phosphate assay [59].

### 3.4. Liposome Aggregation

Aggregation of lipid vesicles was monitored by visible absorbance measurements. The buffer used was PBS, pH 7.2. Peptides (5, 10, 20 and 40 μM) in PBS solutions were added to a suspension of 400 μM LUVs consisting of PC, PC:SM (2:1, *w*/*w*) and PC:CH (2:1, *w*/*w*). Increased absorbance indicated aggregation of liposomes. Absorbance was measured at 405 nm using a microplate Autoreader before and after the addition of peptides [60].

### 3.5. Trp Fluorescence and Acrylamide Quenching Assay

Fluorescence emission spectra of Trp residue in peptides were monitored in the presence of PC, PC:SM (2:1, *w*/*w*) and PC:CH (2:1, *w*/*w*) LUVs. In these fluorescence studies, LUVs were used to minimize differential light scattering effects [61]. Trp fluorescence measurements were carried out using a spectrofluorometer. Each peptide was added to 1 ml of 200 μM liposomes, and the peptide:liposome mixture (a molar ratio of 1:100) was allowed to interact at 25 °C for 10 min. Fluorescence was measured at an excited wavelength of 280 nm an emission wavelength ranging from 300 to 400 nm.

Fluorescence quenching experiments were conducted using acrylamide as a quencher. The acrylamide concentration in the cuvette ranged from 0.04 to 0.20 M. The effect of acrylamide on the fluorescence of each peptide was analyzed using a Stern-Volmer Equation:

(1) $$F_{0}/F=1+K_{\text{SV}}\;(Q)$$

where *F*_0_ and *F* represent the fluorescence intensities in the absence and presence of acrylamide, respectively, *K*_SV_ is the Stern-Volmer quenching constant and (*Q*) is the concentration of acrylamide.

### 3.6. Calcein Leakage from Liposomes

Calcein-entrapped LUVs composed of PC, PC:SM (2:1, *w*/*w*) and PC:CH (2:1, *w*/*w*) were prepared by vortexing the dried lipid in a dye buffer solution (70 mM calcein, PBS, pH 7.4). The suspension was freeze-thawed in liquid nitrogen for nine cycles, after which the calcein-entrapped vesicles were separated from free calcein by gel filtration chromatography on a Sephadex G-50 column. Entrapped LUVs in a suspension containing 100 μM lipids were then incubated with various concentrations of peptide (0.625–10 μM) for 25 min. The fluorescence of released calcein was assessed using a spectrofluorometer at an excitation wavelength of 480 nm and an emission wavelength of 520 nm. Complete (100%) release was achieved by the addition of 0.1% Triton X-100. Spontaneous leakage was determined to be negligible. All experiments were conducted at 25 °C, and the apparent percentage of released calcein was calculated according to the following Equation [62]:

(2) $$\text{Release }(\%)=100\times (F-F_{\text{o}})/(F_{\text{t}}-F_{\text{o}})$$

where *F* and *F*_t_ represent the fluorescence intensity before and after the addition of detergent, respectively, and *F*_o_ represents the fluorescence of intact vesicles.

### 3.7. Circular Dichroism (CD) Spectroscopy

The CD spectra were recorded on a Jasco 810 spectropolarimeter (Jasco, Tokyo, Japan) equipped with a temperature control unit using a 0.1-cm path-length quartz cell at 25 °C between 190 and 250 nm. The CD spectra were measured for peptide samples (50 μM) that were dissolved in PBS (pH 7.2) containing 1 mM PC, 1 mM PC:SM (2:1, *w*/*w*) vesicles or 1 mM PC:CH (2:1, *w*/*w*) vesicles. CD data represent the average value from three separate recordings, with four scans per sample. All CD spectra shown had corresponding peptide-free solvent baselines subtracted. The results are expressed in terms of molar residue CD.

## 4. Conclusions

Among (KW)*_n_* peptides, only (KW)_5_ induced permeation/aggregation of PC vesicles alone or in combination with CH or SM. Eukaryotic membrane aggregation induced by (KW)_5_ was dependent on both the presence of a more hydrophobic patch, as well as its ability to self-aggregate in aqueous solution compared to (KW)_4_. Therefore, (KW)_5_ is not a selective peptide against bacterial membranes compared to (KW)_4_.

## Figures and Tables

**Table 1:** *K*_SV_ parameters of peptides at 2 μM in PBS (pH 7.2) or in the presence of 200 μM PC, 200 μM PC:SM (2:1, *w*/*w*) and 200 μM PC:CH (2:1 *w*/*w*) LUVs.

Peptides	*K*_SV_ (M^−1^) a		

	PC	PC:SM (2:1,*w*/*w*)	PC:CH (2:1 *w*/*w*)
(KW)_3_	7.5	7.1	6.8
(KW)_4_	7.2	6.8	6.2
(KW)_5_	3.2	2.5	1.7

Notes:

a *K*_SV_ is the Stern-Volmer constant; *K*_SV_ (M^−1^) was determined from the Stern-Volmer Equation *F*_0_/*F*_1_ = 1+ *K*_SV_ (*Q*), where *Q* is the concentration of quencher (acrylamide); Concentration of the quencher varied from 0.04 to 0.20 M.
